# Few Differences in the Externalizing and Criminal History of Young Violent Offenders With and Without Autism Spectrum Disorders

**DOI:** 10.3389/fpsyt.2019.00911

**Published:** 2019-12-17

**Authors:** Björn Hofvander, Sophie Bering, André Tärnhäll, Märta Wallinius, Eva Billstedt

**Affiliations:** ^1^Child and Adolescent Psychiatry, Department of Clinical Sciences, Lund, Faculty of Medicine, Lund University, Lund, Sweden; ^2^Centre of Ethics, Law and Mental Health, Department of Psychiatry and Neurochemistry, Institute of Neuroscience and Physiology, The Sahlgrenska Academy at University of Gothenburg, Gothenburg, Sweden; ^3^Gillberg Neuropsychiatry Centre, Institute of Neuroscience and Physiology, University of Gothenburg, Gothenburg, Sweden

**Keywords:** autism spectrum disorder, externalizing behaviour, attention deficit disorder with hyperactivity, conduct disorder, psychopathy, crime, violence

## Abstract

Autism spectrum disorders (ASDs) are known to be associated with an increased risk of aggression and challenging behavior. In this study, we have mapped the externalizing history of a nationally representative cohort of young violent offenders with ASD, compared with offenders without ASD. Two hundred and sixty-nine violent offenders were assessed for prevalence of ASD, and participated in a thorough assessment of previous externalizing problems and criminal history. Twenty-six offenders met consensus clinical DSM-IV criteria for ASD and they were compared to offenders without ASD from the same cohort. Overall, we found a very high prevalence of externalizing and antisocial behaviors in the history of these offenders and there were few differences between the groups. Placements in foster homes were overrepresented in the ASD group and the ASD-offenders had significantly more often been diagnosed with a neurodevelopmental disorder (i.e. ASD or ADHD) by a clinician before the study. At index conviction, ASD offenders were overrepresented in sex crimes with a child victim. Though offenders without ASD had more previous convictions, in particular drug crimes, we found no difference in terms of total number of prosecuted crimes. Substance use disorders were more common among offenders without ASD. The ASD offenders scored higher compared to the non-ASD offenders on the Affective facet of the Psychopathy Checklist-Revised (PCL-R) but there were no differences in terms of total PCL-R scores. Our results provide important knowledge of the developmental history of offenders with ASD. Though this is a small and atypical phenotype it poses significant challenges to the criminal justice system and we need to understand more of it to be able to prevent these individuals from committing crimes but also to provide a fair judicial treatment, to assess exculpatory factors and improve our forensic treatment models.

## Introduction

Violent and criminal behavior in individuals with Autism Spectrum Disorder (ASD) has often been considered a sensitive topic to discuss, particularly in individuals with so-called high-functioning ASD (i.e., without a general intellectual disability) (1). Their lack of reciprocity, social naivety, compulsivity and resistance to change has been raised as possible spectrum characteristics that could explain instances of criminal behavior. However, researchers have expressed worries that reports of serious offences may lead to unnecessary anxiety on the part of parents and to stigmatization of people with ASD (2). There is a lack of research which try to disentangle the empirical background of persistent violent and criminal behavior among individuals with ASD in the general population (e.g. 3, 4).

Almost two decades ago, Moffitt and colleagues (5) suggested a common neurodevelopmental basis for ASD and childhood-onset antisocial behavior. In later support of this, twin studies have shown that the same genetic and environmental factors that are linked to ASD also influence the development of oppositional and conduct problems (6) and autism-like social interaction problems are implicated as among the strongest predictors of conduct problems (7). In a study of childhood arrestees, delinquent behavior was positively associated with autism symptoms, even after adjustment for externalizing disorders, i.e. ADHD and conduct disorder (CD) (8). Studying a large population-based record-linkage cohort, Heeramun and colleagues (4), replicated this increased risk of violent offending among individuals with ASD, but contrary to Geluk and colleagues, when controlling for ADHD and CD this relationship disappeared. Other studies have shown that persistently disruptive children often have autism behavioral traits, and as many as one third might meet criteria for an ASD (9, 10), but only in a small minority of these children, their ASD is detected by the psychiatric services. Similarly, in clinical groups of children with ASD, conduct problems are common, though seldom noted in their medical record. Particularly atypical autism seems to be overrepresented in children with CD (e.g., 11).

Childhood psychosocial adversities and maladjustment have previously been linked to a number of negative outcomes over the life time, including antisocial behaviors (12, 13). Among children with ASD, Howlin and Clements (14) found abuse to be associated with more behavioral difficulties, including aggression, compared to typically developed children. Similarly, Allely et al. (15) have described the presence of a broad range of adverse contextual factors among individuals with ASD who commit serious violent crimes.

Certain psychiatric disorders can increase the risk of violent behavior (16–18), with a particularly strong relationship between substance abuse and repeated violent criminality (e.g. 19). Likewise, this relationship seems to correspond for individuals with ASD (3, 20). Substance use-related problems have traditionally been considered rare in ASD, but recently a review pointed to large variability in different studies (21) and Butwicka and colleagues (22) even showed an increased risk for substance use related problems in a population-based cohort study, without a link to ADHD. From earlier studies in clinical settings (e.g. 23), we know that substance use disorder (SUD) rates can be substantial, and that comorbid ADHD could be a contributing factor to increased SUD in ASD.

Among adults with ASD, there are numerous case reports of serious and persistent offending behavior (e.g., 24–27). Though there are some reports of non-violent criminality (e.g., 28), violent crimes seem clearly overrepresented in the literature. Within the category of violent crime and ASD, arson (e.g., 29, 30) and sexual offence (e.g. 31–33) have gained specific attention. However, the quality of research in this area is generally low and often hampered by the fact that the prevalence of ASD, most probably due to clinical practice and diagnostic criteria, has risen dramatically over the last 20 years, making long term follow-up difficult. Several authors have noted that we need to find out more about the relation between ASD and criminal behavior (34, 35).

To summarize, there has been a vivid debate on the criminal propensity of individuals with ASD and a striking difference in results between population-based studies and criminal cohort studies. In cases where ASD is connected to criminal behavior there are different views as to which kinds of antisocial acts these individuals commit. To address this issue, the present study compares violent offenders with and without ASD on a range of measures of externalizing and criminal behavior over their life-course in an effort to map the developmental history of aggressive and antisocial behaviors in ASD.

## Methods

### Procedure

The study was approved by the Research Ethics Committee at Lund University. All inmates in the participating prison facilities received oral and written information about the study from a prison staff and those that agreed to participate in the study provided written informed consent.

Participants were consecutively assessed according to a pre-set protocol. The clinical assessments, which were performed by experienced clinical psychologists who had a special training in the instruments used, were conducted during a full day session. Before the assessment started, the psychologist had read all file information, including prison health care journals, detailed reports on previous living circumstances and criminal history, and incidents during ongoing sanction, available from the Swedish Prison and Probation Service.

The participants were also given the opportunity to receive feedback on the preliminary results from the assessments. Participants showing indications of severe psychopathology were given the opportunity to be referred to the prison’s psychiatrist for further assessment and treatment. A small monetary compensation for time spent in the study was provided (SEK 200, approximately $22).

### Participants

Participants (n = 269) consisted of male violent offenders recruited from the Development of Aggressive Antisocial Behavior Study (DAABS). The DAABS is a nationally representative cohort of all young adult male offenders (aged 18–25 years) convicted of hands-on violent (including sexual) offenses and imprisoned in one out of nine prisons (low to high security levels) in the Western Region of the Swedish Prison and Probation Service between March 2010 and July 2012. The participation rate was 71%. Detailed descriptions of the cohort are provided in previous publications (36–38). In the total DAABS cohort, 26 participants (10%) met criteria for an ASD at the clinical assessment (Autistic disorder n = 2, Asperger´s disorder n = 18, Pervasive developmental disorder not otherwise specified n = 6). The remaining 243 participants did not have an ASD and were used as a comparison group in this study. There were no statistically differences between the groups in terms of age (ASD: 21.6 years (19.0-25.9), non-ASD: 22.2 years (18.6-25.9), p = 0.090). Two clinical assessments were prematurely ended, because of the participants’ clinical conditions, and on some variables there was insufficient or opposing information, which resulted in missing data.

### Diagnostic Evaluation

Participants were assessed for lifetime and current psychiatric disorders by a structured interview protocol based on the Structured Clinical Interview for DSM-IV Axis I Disorders (SCID-I) and the SCID-II. For the disorders not covered in the SCID (e.g. developmental disorders, impulse control disorders and sexual disorders), an amendment including a lifetime DSM-IV (39) symptom checklist of individual criteria or symptom definitions was added.

For assessment of ASD, the Asperger Syndrome/high functioning autism Diagnostic Interview (ASDI), (40) was used, which is a combined interview and observation schedule for clinical assessment. All participants were also asked about the presence of atypical sensory perception, a commonly reported symptom in ASD. When possible, a collateral interview (the Autism-Tics. ADHD and other Comorbidities inventory/A-TAC, 41) was performed to obtain the developmental history of the participants. In many cases it turned out extremely difficult to get in touch with their families and it was only in a minority of cases an interview could be performed. The A-TAC was used in six ASD cases (23.1%) and in thirty (12.3%) of the non-ASD cases. For participants potentially meeting diagnostic criteria for an ASD-disorder, the team tried to perform an in-depth autism spectrum examination, including either a “Diagnostic Interview for Social and Communication disorders” (the DISCO), (42) with parents/caregivers or an “Autism Diagnostic Observation Schedule” (ADOS), (43) with the participant. The DISCO was used in one ASD case (3.8%) and the ADOS in three cases (11.5%).

Final diagnostic decisions were based on all the available information, provided by the files, registers, clinical and collateral interviews, self-rating questionnaires and the clinical impression of the respondent during the 6–7 hours assessment, in consensus by the clinical psychologist and a senior clinician and researcher (EB or BH), in accordance with the LEAD-principle (44). Comorbidity between ADHD and ASD was allowed in order to account for overlap between the two conditions. In accordance with the DSM-IV, age at onset for symptoms of CD was specified and categorized as early onset (age ≤10) or late onset (age >10).

### Previous Externalizing and Criminal Behaviors

Detailed information on the psychosocial background, including externalizing behavior, and criminal history but also previous placements in foster homes and institutions was collected by a structured protocol. In Sweden, out of home placements could be the result of either a destructive behavior of the child or serious maltreatment and abusive conditions in their homes, not seldom a combination of both. The protocol also covered in-depth information on the index offence, divided into the following categories; murder/manslaughter, robbery, assault, sexual offence with adult victim, sexual offence with child victim, and other violent crimes.

Since the availability of official and objective crime data on the participants could vary at the time of assessment, complete official register-based criminal history was collected from the Crime Register, held by the National Council of Crime Prevention, from age of criminal responsibility until the index conviction at inclusion in the DAABS The collected data included all offences defined in the Swedish Penal Code (45:700), Narcotic Drugs Punishment Act (46:64) and the law on punishment of certain traffic offences (47:649), including court convictions, order of summary punishment and omission of prosecution.

Criminal history, including information from the crime register, was divided into six categories: violent offenses (e.g. murder/manslaughter, assault, unlawful threat, robbery, and arson) following Falk and colleagues (19), and the remaining, i.e. sexual offenses, drug-related offenses, property offenses (collapsing theft and vandalism, excluding robbery), traffic violations, and fraud following the definitions of the National Council of Crime Prevention (48). All crimes included attempted and aggravated forms.

### Psychopathy

Psychopathy was measured through the Psychopathy Checklist-Revised (PCL-R, 49), consisting of 20 items rated on a three-point scale (0 = does not apply, 1 = may apply or applies in some respects, 2 = does apply), with total scores ranging from 0 to 40. Though a cut-off score of 30 is often used for assessment and research purposes, Cooke and colleagues (1999, 2005) have proposed a cut-off of 25 point for European prisoners. The offenders were assessed based on all information available from interviews, observations, and files. Analyses utilized the four-facet structure (Interpersonal, Affective, Lifestyle, Antisocial).

### Statistical Analysis

Data were analyzed using SPSS 25 (SPSS, Chicago, IL, USA) software using two-tailed p-values. The level of significance was set at p < 0.05. Descriptive data were expressed in terms of mean values and standard deviations. Between-group differences for categorical data were analyzed using *χ* *^2^* with Fisher´s exact test when n < 5, Phi-values and odd ratios are also reported. For continuous data, we calculated effect sizes using standard mean differences (Cohen’s *d*) for t-test comparisons. Due to the exploratory nature of the study, multiple comparisons were allowed with no adjustments.

## Results

### Background and Index Conviction Characteristics

As seen in **Table 1**, placements in foster homes during upbringing were overrepresented in the ASD-offenders (OR = 2.275, CI = 0.990–5.224, p < .05), but not in institutions. The ASD-offenders had significantly more often been diagnosed with a neurodevelopmental disorder (i.e. ASD or ADHD) by a clinician before participating in the DAABS (ADHD: OR = 4.417, CI = 1.836–10.624, p < .001; ASD: OR = 18.968, CI = 4.236–84.947, p < .001).

**Table 1:** Background and index crime characteristics.

	ASD N (%)	No ASD N (%)	Phi	OR (95% CI)
Placements foster home	11 (42.3)	59 (24.4)	0.121*	2.275 (0.990–5.224)
Placements institutions	10 (38.5)	93 (38.6)	−0.001	0.995 (0.433–2.285)
Previously diagnosed neurodevelopmental disorder				
ADHD	10 (38.5)	30 (12.4)	0.216***	4.417 (1.836–10.624)
ASD	5 (19.0)	3 (1.2)	0.313^a^***	18.968 (4.236–84.947)
				
Murder/manslaughter	2 (7.7)	11 (4.6)	0.043^a^	1.742 (0.365–8.327)
Robbery	11 (42.3)	87 (36.1)	0.038	1.298 (0.571–2.951)
Assault	8 (30.8)	108 (44.8)	−0.084	0.547 (0.229-1.307)
Other violent crimes	9 (34.6)	109 (45.2)	−0.063	0.641 (0.275–1.495)
Sex with adult victim	0 (0)	16 (6.6)	−0.083^a^	0.934 (0.903–0.966)
Sex with child victim	4 (15.4)	10 (4.1)	0.149^a^*	4.200 (1.216–14.503)

^a^Fisher’s Exact Test; ***p < .001, *p < .05.

In terms of index conviction, only sex crime against a child victim differentiated ASD offenders from non-ASD offenders (OR = 4.200, CI = 1.216–14.503, p < .05) among the six violent index crime categories.

### Externalizing Disorders

Among the early onset externalizing disorders identified at the DAABS assessment (**Table 2**), we found no differences between the groups. As adults, only substance use disorder (OR = 0.368, CI = 0.148–0.912, p < .05) differed between the two groups and was less common in the non-ASD group.

**Table 2:** Externalizing disorders and previous convictions.

	ASD N (%)	No ASD N (%)	Phi (ϕ)	OR (95% CI)
Conduct disorder, any onset	22 (88.0)	187 (77.3)	0.076	2.157 (0.622–7.477)
Conduct disorder, childhood onset	10 (38.5)	63 (26.0)	0.091	1.894 (0.810–4.432)
ADHD, childhood	19 (73.1)	150 (62.2)	0.083	1.921 (0.740–4.988)
ADHD, adulthood	13 (50.0)	102 (42.3)	0.057	1.476 (0.647–3.369)
ADHD and conduct disorder, any	16 (66.7)	128 (53.1)	0.078	1.766 (0.728–4.281)
Antisocial personality disorder	13 (52.0)	156 (64.5)	−0.075	0.597 (0.261–1.366)
Substance use disorder, any	18 (69.2)	208 (86.0)	−0.136*	0.368 (0.148–0.912)
	**ASD M (SD)**	**No ASD M (SD)**		**SMD** **^a^** ** (95% CI)**
Age at first conviction	17.1 (2.1)	17.0 (2.3)		0.04 (−1.15–0.97)
Number of convictions	3.0 (1.8)	4.0 (2.4)		0.42 (−0.10–2.10)*
Number of crimes total	13.5 (10.1)	18.5 (16.4)		0.32 (−2.33–12.47)
Number of violent crimes	4.2 (4.3)	5.3 (4.5)		0.26 (−0.91–3.23)
Number deadly violent crimes	0.0 (0.2)	0.1 (0.2)		0.04 (−0.09–0.11)
Number of sex crimes	0.5 (2.0)	0.2 (0.8)		0.2 (−0.74–0.20)
Number of drug crimes	1.6 (3.1)	3.8 (4.6)		0.48 (0.07–4.21)*
Number of property crimes	2.9 (3.1)	3.4 (4.9)		0.09 (−1.76–2.66)
Number of traffic crimes	0.7 (2.0)	2.5 (5.3)		0.35 (−0.57–4.19)
Number of fraud crimes	1.0 (2.2)	0.7 (1.6)		0.15 (−1.05–0.53)

^a^Cohen’s d; *p < .05.

**Table 2** also reports official data from the national Crime Register, including the index conviction at inclusion in the study. The non-ASD offenders had more convictions (SMD = 0.42, CI = −0.10–2.10, p < .05) but there was no difference in terms of number of crimes. When looking at the specific kinds of crimes, the only crime category that stood out was drug crimes (SMD = 0.48, CI = 0.07–4.21, p < .05), where non-ASD offenders were more often represented.

### Psychopathy

In **Table 3**, PCL-R scores are presented. The only facet where there was a significant difference (SMD = 0.53, CI = −2.24 to −0.15, p < .05) was the Affective facet.

**Table 3:** Scores on the Psychopathy Checklist-Revised.

	ASD M (SD)	No ASD M (SD)	SMD^a^ (95% CI)
PCL-R total score	17.7 (7.5)	17.7 (6.9)	0.02 (−3.12–3.36)
Interpersonal facet	1.2 (1.6)	1.0 (1.4)	0.19 (−0.93–0.39)
Affective facet	4.3 (2.2)	3.1 (2.2)	0.53 (−2.24 to −0.15)*
Lifestyle facet	6.0 (2.9)	6.5 (2.6)	0.21 (−0.66–1.76)
Antisocial facet	5.6 (2.8)	6.4 (2.9)	0.29 (−0.51–2.15)

^a^Cohen’s d; *p < .05.

## Discussion

There is a stark difference between population-based studies of individuals with ASD, studies which does not seem to show an increased risk of criminal offending, and the heterogenous literature on identified offenders, where ASD is clearly overrepresented. The prevalence of ASD in prisons and other secure institutions is considerable. Over the last 20 years, several papers have been published showing that between 10 and 20 percent meet criteria for ASD in these settings (36, 50–54). There are studies showing that risk factors for persistent criminality and violence seem to be the same for individuals with as without ASD (e.g. 4, 55, 56) but there have been few studies on the antecedents of adult violent criminality in terms of life history of externalizing and criminal behavior in offenders with ASD.

The present study compared violent offenders with and without ASD on a range of externalizing behaviors, violence and crime-related variables. We found few differences between the groups. In terms of characteristics of the index offence, the ASD group was overrepresented among the sex offenders with a child victim, an offender category where a link to ASD has previously been suggested (e.g. 57). However, we found no differences in terms of life-time convictions for sexual offences. The overall picture of criminal history among these offenders pointed to more similarities than differences. In this context it is crucial to remember that these 26 individuals with ASD seem to represent a very complex clinical phenotype. The whole cohort was characterized by a heavy burden of childhood adversities and an early onset of psychosocial problems, as described in earlier publications (38). The ASD group stood out with an even higher percentage of foster home placements (42% vs. 24%). In a Swedish context, this kind of out-of-home placements mirrors either severe problems with harmful and destructive behavior in these children or serious maltreatment and destructive conditions in their homes, not seldom a combination of both. We know that children with disabilities, including ASD, are at heightened risk of maltreatment (58, 59), which in turn is associated with elevated levels of hyperactivity, aggression, and temper tantrums.

Also, the ASD subjects, as well as the non-ASD subjects in this study, were characterized by massive comorbidity, in neurodevelopmental as well as clinical disorders. There are several studies that links the risk of offending in the ASD group to the simultaneous presence of ADHD (4, 60). In our cohort, three out of four ASD individuals (73.1%) met criteria for childhood ADHD, a finding which has been reported previously (36). Childhood onset conduct problems, perhaps the strongest predictor for future criminality, was also very common among the ASD offenders (38.5%) and two-thirds of all ASD subjects (66.77%) had a combination of ADHD and CD.

It is noteworthy that many of the ASD offenders had previously been recognized for their abnormal developmental, and, in most cases, received an ADHD diagnosis, but in only 19% of the cases an ASD diagnosis. A late diagnosis of ASD have been linked to several contextual factors, including adversity (61) and low socio-economic status (62). We also know that an early identification of ASD is critically important to improve health, level of functioning and wellbeing but we need more studies to find out if it affects the risk of criminality as well.

Lifetime prevalence of SUDs was very high in both groups. Almost 70% of the ASD offenders met criteria for a substance use disorder, though the risk for developing these disorders was even higher in the non-ASD group. As far as we know, the study by Långström and colleagues (3) is the only one that has linked SUDs to an increased risk of violent offending in ASD. Of course, this study cannot replicate these findings but it is valuable to describe the high prevalence of SUDs among these young violent offenders with ASD.

In the official crime register, it was evident that though the non-ASD group had more convictions (p < .05), no difference was found in terms of total number of crimes, violent, sexual, property, traffic or fraud crimes. The non-ASD group was convicted of more drug crimes, which correlates to their higher prevalence of substance use disorders.

The relationship between ASD and psychopathy has been discussed for some time. Frith (63) suggested deficient empathy as a key component in both ASD, and psychopathy, but later research seem to favor the hypothesis that individuals with ASD primarily lack cognitive empathy, while “psychopaths” are low in emotional empathy (1). Despite this interest, there are few empirical studies of individuals representing the so called “double-hit”, i.e. ASD individuals that have additional impairments in empathy and response to distress of other people that are not part of the ASD core symptomatology itself (64). In our study we found scores in both groups comparable to other European prison studies (65) and total scores did not differ between the two groups. However, we noticed a higher score on the Affective facet in the ASD group (p < .05). This facet describes lack of remorse or guilt, shallow affect, callous traits or lack of emotional empathy and failure to accept responsibility for their own actions. In a sample of adolescents with ASD, Carter Leno and colleagues (66) found callous-unemotional traits above the cut-off in 51% of their study group and it was associated with the same deficit in fear recognition previously reported in typically developing samples. This high score was not related to the amount of conduct problems in their group. We still don´t know if cognitive impairments associated with ASD, e.g. deficient theory of mind, increase the risk of developing these traits, perhaps in the presence of maltreatment and suboptimal parenting. In our study, we did not measure the presence of alexithymia, i.e. impaired ability to reflect on and report own emotions. It is possible that alexithymia could mediate some of the variance in the Affective facet.

An obvious limitation in the current study was the small sample and the large number of analyses performed, increasing the risk of type I and II errors. We justify these analyses with the importance of describing this particularly sensitive phenotype in order to be able to identify these individuals much earlier in their development. Relevant and fine-grained data will not be possible to collect through population-based studies and this study represents 20% of the underlying prison population of Sweden (38). Conducting clinical assessments in a prison setting also presents certain challenges, and in many cases parents were not able to give a developmental history of the participants. We applied the LEAD principle in all our diagnostic assessments, still considered to represent the gold standard for psychiatric assessments, and used multiple sources of information about the health and functioning of the participants, present as well as historical.

## Data Availability Statement

The datasets generated for this study are available on request to the corresponding author.

## Ethics Statement

The studies involving human participants were reviewed and approved by Research Ethics committee at Lund University (Dnr: 2009/405). The patients/participants provided their written informed consent to participate in this study.

## Author Contributions

BH and EB conceived and designed the study. EB, MW, and BH obtained the data. BH drafted the initial manuscript with contributions from SB, MW, AT and EB. BH did all the analyses. Finally, all the authors critically revised the manuscript and approved the final version.

## Funding

This work was supported by the Department of Research and Development, Region Kronoberg, the Regional Forensic Psychiatric Clinic in Växjö, Sweden, the Swedish Prison and Probation Service, Södra sjukvårdsregionen, and Region Skåne and Lund University under the ALF-agreement. 

## Conflict of Interest

The authors declare that the research was conducted in the absence of any commercial or financial relationships that could be construed as a potential conflict of interest.

## References

[1] Hofvander B Offenders with autism spectrum disorder. In: Beech AR Carter AJ Mann RE Rotshtein P, editors. The Wiley Blackwell Handbook of Forensic Neuroscience. John Wiley and Sons Ltd.: Hoboken (2018). p. 273–0. 10.1192/bjpo.bp.116.003889

[2] Howlin P Legal issues. In: Howlin P, editor. Autism and Asperger syndrome: Preparing for adulthood, 2nd ed Routledge: London/New York (2004). p. 300–2. 10.1007/BF02179372

[3] Långström N Grann M Ruchkin V Sjöstedt G Fazel S Risk factors for violent offending in autism spectrum disorder. a national study of hospitalized individuals. J Interpers Violence (2009) 24:1358–70. 10.1542/peds.2016-1817 18701743

[4] Heeramun R Magnusson C Hellner Gumpert C Granath S Lundberg M Dahlman C Autism and convictions for violent crimes: population-based cohort study in Sweden. J Am Acad Child Adolesc Psychiatry (2017) 56:491–7. 10.1002/9781118650868.ch11 28545754

[5] Moffitt TE Caspi A Rutter M Silva PA Sex Differences in Antisocial Behavior: Conduct Disorder, Delinquency and Violence in the Dunedin Longitudinal Study. Cambridge University Press: Cambridge (2001). 10.1016/j.rasd.2011.09.003

[6] Lundström S Chang Z Kerekes N Gumpert CH Rastam M Gillberg C Autistic-like traits and their association with mental health problems in two nationwide twin cohorts of children and adults. Psychol Med (2011) 41:2423–33. 10.1177/0886260508322195 21426604

[7] Kerekes N Lundström S Chang Z Tajnia A Jern P Lichtenstein P Oppositional defiant- and conduct disorder-like problems: neurodevelopmental predictors and genetic background in boys and girls, in a nationwide twin study. PeerJ (2014) 2:e359. 10.1080/14789940903174170 24795851 PMC4006222

[8] Geluk CA Jansen LM Vermeiren R Doreleijers TA van Domburgh L de Bildt A Autistic symptoms in childhood arrestees: longitudinal association with delinquent behavior. J Child Psychol Psychiatry (2012) 53:160–7. 10.1177/1362361301005001006 21884523

[9] Donno R Parker G Gilmour J Skuse DH Social communication deficits in disruptive primary-school children. Br J Psychiatry (2010) 196:282–9. 10.1001/archgenpsychiatry.2008.537 20357304

[10] Gilmour J Hill B Place M Skuse DH Social communication deficits in conduct disorder: a clinical and community survey. J Child Psychol Psychiatry (2004) 45:967–78. 10.1186/1471-244X-10-112 15225339

[11] de Bruin EI Ferdinand RF Meester S de Nijs PFA Verheij F High rates of psychiatric co-morbidity in PDD-NOS. J Autism Dev Disord (2007) 37:877–86. 10.1016/j.avb.2018.01.007 17031447

[12] af Klinteberg B Almquist Y Beijer U Rydelius PA Family psychosocial characteristics influencing criminal behavior and mortality—Possible mediating factors: A longitudinal study of male and female subjects in the Stockholm Birth Cohort. BMC Public Health (2011) 11:756. 10.1186/1471-2458-11-756 21962152 PMC3198708

[13] Schilling EA Aseltine RH Jr Gore S Adverse childhood experiences and mental health in young adults: a longitudinal survey. BMC Public Health (2007) 7:30. 10.1007/s11195-013-9286-8 17343754 PMC1832182

[14] Howlin P Clements J Is it possible to assess the impact of abuse on children with pervasive developmental disorders? J Autism Dev Dis (1995) 25:337–54. 10.3109/08039488.2013.780259 7592248

[15] Allely CS Minnis H Thompson L Wilson P Gillberg C Neurodevelopmental and psychosocial risk factors in serial killers and mass murderers. Aggress Violent Behav (2014) 19:288–1. 10.1016/j.avb.2014.04.004

[16] Elbogen EB Johnson SC The intricate link between violence and mental disorder: results from the National Epidemiologic Survey on Alcohol and Related Conditions. Arch Gen Psychiatry (2009) 66:152–61. 10.1007/s00127-013-0783-y 19188537

[17] Fazel S Wolf A Chang Z Larsson H Goodwin GM Lichtenstein P Depression and violence: A Swedish population study. Lancet Psychiatry (2015) 2:224–32. 10.1017/CBO9780511526770 PMC4520382 26236648

[18] Chang Z Larsson H Lichtenstein P Fazel S Psychiatric disorders and violent reoffending: a national cohort study of convicted prisoners in Sweden. Lancet Psychiatry (2015) 2:891–0. 10.1034/j.1600-0447.2003.01354.x PMC4629414 26342957

[19] Falk O Wallinius M Lundström S Frisell T Anckarsäter H Kerekes N The 1% of the population accountable for 63% of all violent crime convictions. Soc Psychiatry Psychiatr Epidemiol (2014) 49:559–71. 10.1016/S2215-0366(14)00128-X PMC3969807 24173408

[20] Newman S Ghaziuddin M Violent crime in Asperger syndrome: the role of psychiatric comorbidity. J Autism Dev Disord (2008) 38:1848–52. 10.1371/journal.pone.0137475 18449633

[21] Arnevik EA Helverschou SB Autism spectrum disorder and co-occurring sub- stance use disorder – a systematic review. Subst Abuse (2016) 10:69–5. 10.4137/SART.S39921 PMC4990150 27559296

[22] Butwicka A Långström N. Larsson H Lundström S Serlachius E Almqvist C Increased risk for substance use-related problems in autism spectrum disorders: a population-based cohort study. J Autism Dev Disord (2017) 47:80–9. 10.1007/s10803-016-2914-2 PMC5222913 27734228

[23] Hofvander B Delorme R Chaste P Nydén A Wentz E Ståhlberg O Psychiatric and psychosocial problems in adults with normal-intelligence autism spectrum disorders. BMC Psychiatry (2009) 9:35. 10.1016/j.psychres.2010.05.008 19515234 PMC2705351

[24] Baron-Cohen S An assessment of violence in a young man with Asperger’s syndrome. J Child Psychol Psychiatry (1988) 29:351–60. 10.1111/j.1469-7610.1988.tb00723.x 3417810

[25] Mawson D Grounds A Tantam D Violence and Asperger’s syndrome: A case study. Br J Psychiatry (1985) 147:566–9. 10.1097/DBP.0000000000000097 4075056

[26] Murrie DC Warren JI Kristiansson M Dietz PE Asperger’s syndrome in forensic settings. Int J Forensic Ment Health (2002) 1:59–0. 10.1080/14999013.2002.10471161

[27] Wing L Asperger’s syndrome: A clinical account. Psychol Med (1981) 11:115–29. 10.1111/1469-7610.00023 7208735

[28] Chen PS Chen SJ Yang YK Yeh TL Chen CC Lo HY Asperger’s disorder: A case report of repeated stealing and the collecting behaviours of an adolescent patient. Acta Psychiat Scand (2003) 107:73–6. 10.1016/S0165-1781(97)00119-4 12558546

[29] Palermo MT Pervasive developmental disorders, psychiatric comorbidities, and the law. Int J Offender Ther Comp Criminol (2004) 48:40–8. 10.1017/S0033291706008853 14969115

[30] Tantam D Asperger syndrome in adulthood. In: Frith U, editor. Autism and Asperger syndrome. Cambridge University Press: Cambridge (1991). p. 147–3. 10.4088/JCP.08m04635

[31] Sevlever M Roth ME Gillis JM Sexual abuse and offending in autism spectrum disordes. Sex Disabil (2013) 31:189–0. 10.1007/s11195-013-9286-8

[32] ‘t Hart-Kerkhoffs LA Jansen LM Doreleijers TA Vermeiren R Minderaa RB Hartman CA Autism spectrum disorder symptoms in juvenile suspects of sex offenses. J Clin Psychiatry (2009) 70:266–72. 10.1037/lhb0000202 19210944

[33] Mouridsen SE Rich B Isager T Nedergaard NJ Pervasive developmental disorders and criminal behavior. a case control study. Int J Offender Ther Comp Criminol (2008) 52:196–5. 10.1080/14999013.2002.10471161 17615427

[34] Björkly S Risk and dynamics of violence in Asperger’s syndrome: a systematic review of the literature. Aggress Violent Behav (2009) 14:306–12. 10.1016/j.avb.2009.04.003

[35] Mouridsen SE Current status of research on autism spectrum disorders and offending. Res Autism Spectr Disord (2012) 6:79–6. 10.1177/0306624X07302056

[36] Billstedt E Wallinius M Anckarsäter H Hofvander B Neurodevelopmental disorders in young violent offenders: overlap and background characteristics. Psychiatry Res (2017) 252:234–41. 10.1016/j.psychres.2017.03.004 28285251

[37] Hofvander B Anckarsäter H Wallinius M Billstedt E Mental health among young adults in prison: the importance of childhood onset conduct disorder. BJPsych Open (2017) 3:78–4. 10.1186/1471-244X-9-35 PMC5362727 28357134

[38] Wallinius M Delfin C Billstedt E Nilsson T Anckarsäter H Hofvander B Offenders in emerging adulthood: School maladjustment, childhood adversities and prediction of aggressive antisocial behaviors. Law Hum Behav (2016) 40:551–63. 10.1017/S0033291700053332 27243360

[39] American Psychiatric Association (APA). Diagnostic and Statistical Manual of Mental Disorders (DSM-IV). 4th ed text revision. American Psychiatric Press: Washington, DC (2000).

[40] Gillberg C Gillberg IC Rastam M Wentz E The Asperger Syndrome Diagnostic Interview (ASDI): a preliminary study of a new structured clinical interview. Autism (2001) 5:57–6. 10.1111/j.1469-7610.2004.t01-1-00289.x 11708390

[41] Hansson SL Svanstrom Rojvall A Rastam M Gillberg C Anckarsater H Psychiatric telephone interview with parents for screening of childhood autism – tics, attention-deficit hyperactivity disorder and other comorbidities (A-TAC): Preliminary reliability and validity. Br J Psych (2005) 187:262–7. 10.1192/bjp.187.3.262 16135864

[42] Wing L Leekam SR Libby SJ Gould J Larcombe M The diagnostic interview for social and communication disorders: background, inter-rater reliability and clinical use. J Child Psychol Psychiatry (2002) 43:307–25. 10.1080/14789940600589464 11944874

[43] Lord C Risi S Lambrecht L Cook EH Jr. Leventhal BL DiLavore PC The autism diagnostic observation schedule—generic: a standard measure of social and communication deficits associated with the spectrum of autism. J Autism Dev Disord (2000) 30:205–23. 10.1017/S0033291711000377 11055457

[44] Spitzer RL Psychiatric diagnosis: are clinicians still necessary? Compr Psychiatry (1983) 24:399–1. 10.1016/j.comppsych.2004.07.030 6354575

[45] SFS. 700. In: Brottsbalk. Riskdagen: Stockholm (1962).

[46] SFS. 64. In: Narkotikastrafflagen. Riksdagen: Stockholm (1968).

[47] SFS. 649. In: Lag om straff för vissa trafikbrott. Riksdagen: Stockholm (1951).

[48] National Council of Crime Prevention. Kriminalstatistik 2017. Personer lagförda för brott: Stockholm (2018). Author. 10.1007/s10803-008-0580-8

[49] Hare RD PCL-R manual. Multi-Health Systems: Toronto, Ontario, Canada (2003). 10.1016/j.jaac.2017.03.011

[50] Siponmaa L Kristiansson M Jonson C Nyden A Gillberg C Juvenile and young adult mentally disordered offenders: the role of child neuropsychiatric disorders. J Am Acad Psychiatry Law (2001) 29:420–6. 10.1016/0010-440X(83)90032-9 11785613

[51] Söderström H Nilsson T Sjödin AK Carlstedt A Forsman A The childhood-onset neuropsychiatric background to adulthood psychopathic traits and personality disorders. Compr Psychiatry (2005) 46:111–6. 10.1017/CBO9780511526770.005 15723027

[52] Kumagami T Matsuura N Prevalence of pervasive developmental disorder in juvenile court cases in Japan. J For Psychiatr Psychol (2009) 20:974–87. 10.1023/A.1005592401947

[53] Ginsberg Y Hirvikoski T Lindefors N Attention deficit hyperactivity disorder (ADHD) among longer-term prison inmates is a prevalent, persistent and disabling disorder. BMC Psychiatry (2010) 10:112. 10.1192/bjp.187.3.262 21176203 PMC3016316

[54] Young S Gonzalez RA Mullens H Mutch L Malet-Lambert I Gudjonsson GH Neurodevelopmental disorders in prison inmates: comorbidity and combined associations with psychiatric symptoms and behavioral disturbance. Psychiatry Res (2018) 261:109–15. 10.1016/j.psychres.2017.12.036 29291476

[55] Kawakami C Ohnishi M Sugiyama T Someki F Nakamura K Tsujii M The risk factors for criminal behavior in high functioning autism spectrum disorders (HFASDs): a comparison of childhood adversities between individuals with HFASDs who exhibit criminal behavior and those with HFASD and no criminal histories. Res Autism Spectr Disord (2012) 6:949–57. 10.7717/peerj.359

[56] Del Pozzo J Roché MW Silverstein SM Violent behavior in autism spectrum disorders: who’s at risk? Aggress Violent Behav (2018) 39:53–0. 10.1192/bjp.bp.108.061341

[57] Woodbury-Smith MR Clare ICH Holland AJ Kearns A High functioning autistic spectrum disorders, offending and other law-breaking: findings from a community sample. J Forens Psychiatry Psychol (2006) 17:108–20. 10.1016/j.psychres.2017.12.036

[58] Maclean MJ Sims S Bower C Leonard H Stanley FJ O’Donnell M Maltreatment risk among children with disabilities. Pediatrics (2017) 139:e20161817. 10.1192/bjp.147.5.566 28264988

[59] McDonnell CG Boan AD Bradley CC Seay KD Charles JM Carpenter LA Child maltreatment in autism spectrum disorder and intellectual disability: Results from a population-based sample. J Child Psychol Psychiatr (2018) 60:576–84. 10.1017/CBO9780511490057 PMC6458088 30368827

[60] Nore´n Selinus E Molero Y Lichtenstein P Larson T Lundström S Anckarsäter H Heller Gumpert C Childhood Symptoms of ADHD Overrule comorbidity in relation to psychosocial outcome at age 15: a longitudinal study. PLoS One (2015) 10:e0137475. 10.1177/0306624X03257713 26360378 PMC4567137

[61] Berg KL Acharya K Shiu C-S Msall ME Delayed diagnosis and treat- ment among children with autism who experience adversity. J Autism Dev Disord (2018) 48:45–4. 10.1007/s10803-017-3294-y 28864845

[62] Mazurek MO Handen BL Wodka EL Nowinski L Butter E Engelhardt CR Age at first autism spectrum disorder diagnosis: The role of birth cohort, demographic factors, and clinical features. J Dev Behav Pediatr (2014) 35:561–9. 10.1111/jcpp.12993 25211371

[63] Frith U Autism and asperger syndrome. Cambridge University Press: Cambridge, New York (1991). 10.1111/j.1469-7610.2011.02456.x

[64] Rogers J Viding E Blair RJ Frith U Happe F Autism spectrum disorder and psychopathy: shared cognitive underpinnings or double hit? Psychol Med (2006) 36:1789–98. 10.1186/1471-2458-7-30 17018169

[65] Jüriloo A Lauerma H Holmalahti T Tyni S Aarnio J Viitanen P Psychopathic traits in a representative sample of Finnish male prisoners. Nord J Psychiatry (2014) 68:117–22. 10.3109/08039488.2013.780259 23566030

[66] Carter Leno V Charman T Pickles A Jones CR Baird G Happe F Callous–unemotional traits in adolescents with autism spectrum disorder. Br J Psych (2015) 207:392–9. 10.1192/bjp.bp.114.159863 PMC4629071 26382954

